# Bloom-Forming Cyanobacteria Support Copepod Reproduction and Development in the Baltic Sea

**DOI:** 10.1371/journal.pone.0112692

**Published:** 2014-11-19

**Authors:** Hedvig Hogfors, Nisha H. Motwani, Susanna Hajdu, Rehab El-Shehawy, Towe Holmborn, Anu Vehmaa, Jonna Engström-Öst, Andreas Brutemark, Elena Gorokhova

**Affiliations:** 1 Department of Ecology, Environment and Plant Sciences, Stockholm University, Stockholm, Sweden; 2 IMDEA Agua, Alcalá de Henares, Madrid, Spain; 3 Calluna AB, Stockholm, Sweden; 4 ARONIA Coastal Zone Research Team, Novia University of Applied Sciences & Åbo Akademi University, Ekenäs, Finland; 5 Tvärminne Zoological Station, University of Helsinki, Hangö, Finland; 6 Department of Applied Environmental Science, Stockholm University, Stockholm, Sweden; Stazione Zoologica Anton Dohrn, Naples, Italy

## Abstract

It is commonly accepted that summer cyanobacterial blooms cannot be efficiently utilized by grazers due to low nutritional quality and production of toxins; however the evidence for such effects *in situ* is often contradictory. Using field and experimental observations on Baltic copepods and bloom-forming diazotrophic filamentous cyanobacteria, we show that cyanobacteria may in fact support zooplankton production during summer. To highlight this side of zooplankton-cyanobacteria interactions, we conducted: (1) a field survey investigating linkages between cyanobacteria, reproduction and growth indices in the copepod *Acartia tonsa*; (2) an experiment testing relationships between ingestion of the cyanobacterium *Nodularia spumigena* (measured by molecular diet analysis) and organismal responses (oxidative balance, reproduction and development) in the copepod *A. bifilosa*; and (3) an analysis of long term (1999–2009) data testing relationships between cyanobacteria and growth indices in nauplii of the copepods, *Acartia* spp. and *Eurytemora affinis*, in a coastal area of the northern Baltic proper. In the field survey, *N. spumigena* had positive effects on copepod egg production and egg viability, effectively increasing their viable egg production. By contrast, *Aphanizomenon* sp. showed a negative relationship with egg viability yet no significant effect on the viable egg production. In the experiment, ingestion of *N. spumigena* mixed with green algae *Brachiomonas submarina* had significant positive effects on copepod oxidative balance, egg viability and development of early nauplial stages, whereas egg production was negatively affected. Finally, the long term data analysis identified cyanobacteria as a significant positive predictor for the nauplial growth in *Acartia* spp. and *E. affinis*. Taken together, these results suggest that bloom forming diazotrophic cyanobacteria contribute to feeding and reproduction of zooplankton during summer and create a favorable growth environment for the copepod nauplii.

## Introduction

Toxic blooms of filamentous cyanobacteria are proliferating worldwide due to the climate change and eutrophication [1]. These cyanobacteria are commonly considered to impair survival, growth and reproduction of grazers [2]. The negative effects of cyanobacteria on zooplankton are usually related to a combination of (*i*) low nutritional value due to inadequate dietary fatty acid composition [3], [4], (*ii*) production of toxins and feeding deterrents [5], [6], and (*iii*) poor manageability of the colonies [7]. However, there are also studies showing that cyanobacteria have neutral or positive effects on zooplankton egg production and growth [8]–[12]. Application of stable isotopes, fatty acids and DNA-based methods [13]–[16] for zooplankton diet analysis suggest relatively high *in situ* grazing on colony-building cyanobacteria in freshwaters and estuaries, where summer cyanobacterial blooms are a regular feature. One can speculate that when preferred prey is scarce, zooplankton may increase feeding on the abundant cyanobacteria, despite their inadequate biochemical composition and toxicity. We know, for example, from terrestrial ecology that rainforest-dwelling parrots consume toxic foods during dry seasons when other, non-toxic, food is limited [17]. Supporting these contradictory reports, meta-analysis of cyanobacteria effects on various grazers [18], [19] suggest that cyanobacterial effects on biota are multifactorial and species- and system-specific [20].

In the Baltic Sea, summer blooms of diazotrophic filamentous cyanobacteria are dominated by *Aphanizomenon* sp., *Nodularia spumigena* and *Dolichospermum* spp. [21]. Biomass of Baltic mesozooplankton peaks during the same period as that of cyanobacteria, with calanoid copepods being the most important group [22], [23]. The evidence is accumulating that cyanobacterial blooms may have a more important role in the Baltic food webs than previously assumed, and it has been suggested that utilization of cyanobacteria as a food source by zooplankton in this system is underestimated [24]. For example, a strong nitrogen isotopic signal in of various size following summer bloom in the northern Baltic Sea [25], indicates that nitrogen (N) fixed by diazotrophic cyanobacteria is directly or indirectly utilized in the food web. Indeed, using both isotopic signals [26] and cyanobacterial pigments as tracers, copepods in the Baltic proper have been found to assimilate cyanobacteria in measurable quantities [24]. Also, lipid signal in the copepod *Pseudocalanus acuspes* in the southern Baltic Sea indicates that cyanobacteria contribute substantially to its diet during summer [27]. Finally, studies employing DNA-based analysis of stomach content provide unequivocal evidence that Baltic copepods and mysids ingest filamentous cyanobacteria *in situ* even when alternative food is present [14].

Similarly, effects of filamentous cyanobacteria on survival and development are often contradictory, with both negative [12], [28] and positive [9], [10] effects being observed. As a monospecific food, cyanobacteria do not seem to support neither egg production [11], [28], [29] nor egg hatching [28] in copepods, with nodularins and microcystins being the most commonly implicated in these effects. These hepatotoxins have been reported to cause oxidative stress in various invertebrates and fish [30], [31] by increasing formation of reactive oxygen species, decreasing the antioxidant capacity (e.g., inhibiting detoxification enzymes) and oxidation of macromolecules (proteins, lipids and DNA). However, when offered in mixtures with alternative prey, filamentous cyanobacteria may have positive effects on copepod egg production, hatching and juvenile development [9], [11], [32]. Due to this conflict of information, more studies on the occurrence and mechanisms of these effects in ecologically relevant settings are needed.

Here, we explored linkages between Baltic Sea diazotrophic filamentous cyanobacteria and fitness-related responses in copepods: reproductive output, juvenile development and growth, and oxidative status. To evaluate these connections, we conducted a set of interrelated studies in the northern Baltic proper. First, in a field survey, we used a correlative approach to relate reproduction (egg production and their viability) of the copepod *Acartia tonsa* to phytoplankton community structure, with specific focus on the effects of the bloom-forming cyanobacteria. Then, we conducted a follow-up laboratory experiment, where ingestion of the cyanobacterium was measured in *A. bifilosa* using molecular diet analysis and related to the copepod oxidative status, and recruitment. Finally, we analyzed long-term data on growth indices in nauplii of *Acartia* spp. and *Eurytemora affinis* in relation to cyanobacteria bloom intensity.

## Methods

### Ethics Statement

The sampling was conducted within Swedish National Marine Monitoring in the Baltic Sea and no specific permissions were required for the sampling locations of this study. Also, we did not require ethical approval to conduct this research as no animals considered in any animal welfare regulations and no endangered or protected species were involved in either field or experimental studies.

### Summer field survey

#### Study sites

**Figure 1:**
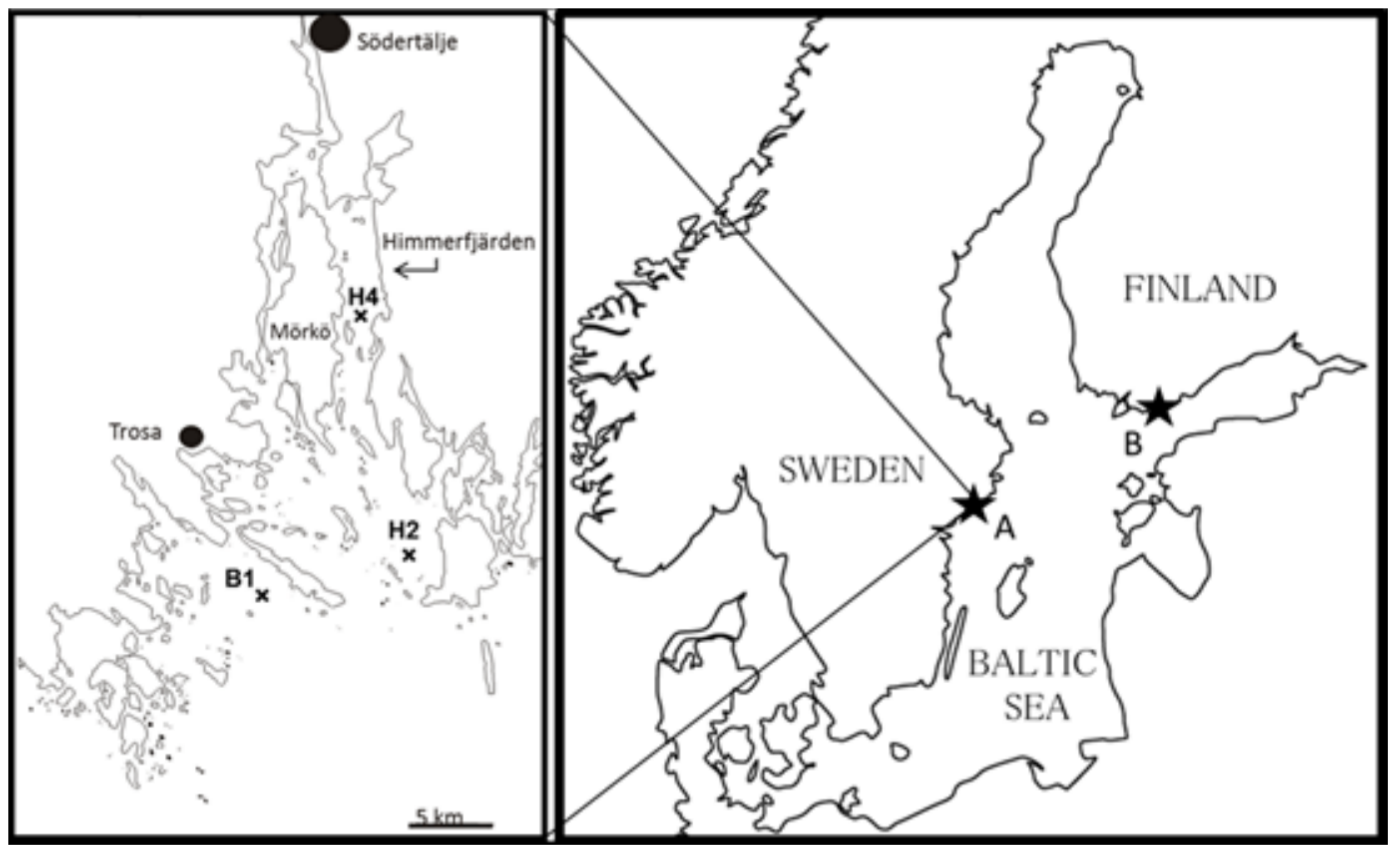
Schematic map over the Baltic Sea and the sampling sites. (A) Himmerfjärden Bay (sampling stations H2 and H4) and a coastal area near Askö laboratory (station B1) in the western part of the northern Baltic proper, where field data for the summer field survey and the long term data (stn H4 and B1) were collected, and (B) Storfjärden Bay, at the entrance to the Gulf of Finland, where study animals for the laboratory experiment were collected.

Sampling was conducted biweekly during cyanobacterial bloom (July 4 to September 26, 2007) at three stations in the north-west Baltic proper (58°49'N, 17°39'E; Figure 1). Two of these stations are located in the Himmerfjärden Bay (stns H2 and H4; SYVAB's marine monitoring program) and stn B1 (Swedish National Marine Monitoring Program, SNMMP) is outside the bay (Figure 1).

#### Sampling

Sea surface temperature (SST) was measured on each sampling occasion. Integrated phytoplankton samples were collected by a hose (inner diameter 19 mm) at stns H2 and H4 (0–14 m) and stn B1 (0–20 m), and preserved with acidic Lugol's solution. Using an inverted microscope (Wild M40), cells counts (>2 µm) and biovolume analysis were conducted according to HELCOM monitoring guidelines [33], [34]; see also http://www.ices.dk/marine-data/Documents/ENV/PEG_BVOL.zip. Based on size measurements, phytoplankton were divided into size fractions of 2–5 µm, 5–15 µm, 15–30 µm and>30 µm (filamentous cyanobacteria excluded); each species of filamentous cyanobacteria was treated as a separate category regardless of size.

Copepods were sampled by vertical hauls (0–10 m) using a WP2 net (diameter 58 cm, mesh size 200 µm) and brought to the laboratory in 20 L insulated containers. Using a wide-mouth pipette, adult *A. tonsa* females were sorted upon arrival to the laboratory; the choice of species was based on relative abundance of copepod species in the samples and availability of adult females. They were incubated individually for 24 h in darkness at ambient (±2°C) temperature, in microplate wells (12 wells; Corning Costar, Corning NY, USA) filled with 5 mL of 6-µm filtered seawater. The mortality of the females during the incubation was <5%.

#### Reproductive variables

The egg production rate (EPR; eggs female^−1^ day^−1^) was recorded by counting number of eggs in each well under a stereomicroscope (Wild Heerbrugg, 6–50×). No crumbled or empty egg shells were found, indicating absence of egg cannibalism. Live females were individually placed in Eppendorf tubes, frozen at −80°C and stored for a few weeks before RNA analysis. For egg viability (EV%; percentage of viable eggs) analysis, all eggs from each well were transferred to a depression slide and stained with TO-PRO-1 iodide (Molecular Probes) [35]. The viable egg production rate (VEPR; viable eggs female^−1^ day^−1^) was calculated by multiplying EV% with EPR.

#### RNA quantification

As a proxy for growth rate, individual RNA content of the females was used [36] measured by the high-range RiboGreen (Molecular Probes, Inc., Eugene, OR, USA) assay after extraction with N-laurylsarcosine followed by RNase digestion [37]; fluorometer FLUOstar Optima (BMG Labtechnologies; 485 nm for excitation and 520 nm for emission) and black flat bottom microplates (Greiner Bio-One GmbH) were used. Before the analysis, the females (PL, prosome length mm) were measured using an inverted microscope (80×; Wild 40, Heerbrugg) with an ocular micrometer.

### Laboratory experiment

#### Study animals and algal cultures

In August 2010, copepods were collected, with a 150 µm plankton net in the Storfjärden Bay, Western Finland (59°51'20''N, 23°15'42''E; Figure 1). Adult females and males of *Acartia bifilosa* which dominated the copepod community during the study period and area, were gently sorted under a stereo microscope and placed in 1.2 L bottles. Cultures of the green alga *Brachiomonas submarina* (strain TV15, collection of Tvärminne Zoological Station, University of Helsinki) and the cyanobacterium *Nodularia spumigena* (strain AV1 obtained from Prof. K. Sivonen, University of Helsinki) were grown under irradiance of 13.7 µmol photons m^−2^ s^−1^ for 16 h a day, and at 18°C, in f/2 medium (without silica) and Z8 (without nitrogen), respectively [38].

#### Experimental set-up

**Figure 2:**
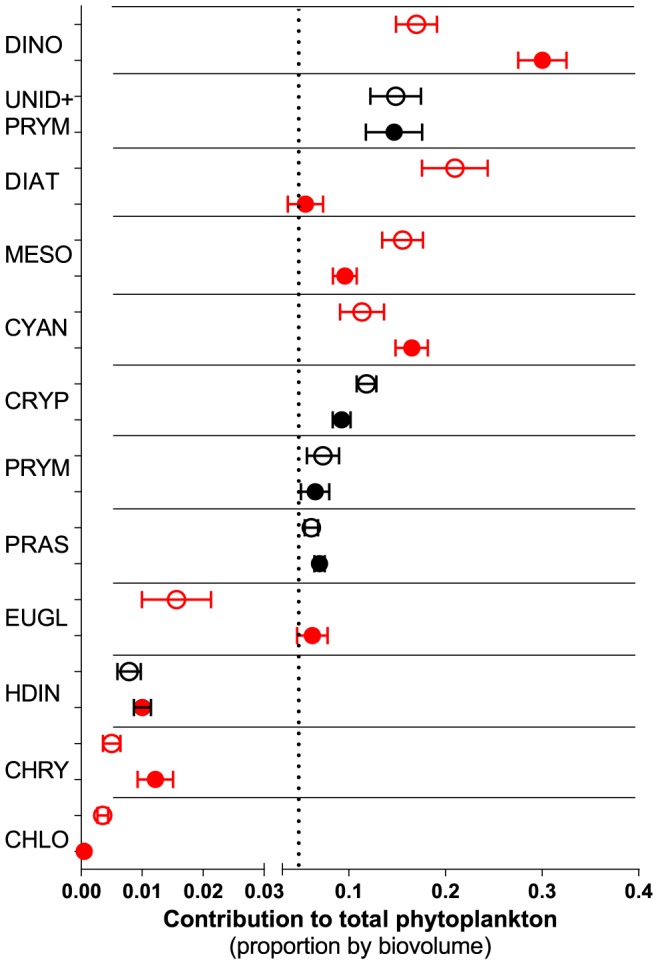
Contribution of the main taxonomic groups to the phytoplankton communities (by biovolume) at stations B1 (closed symbols) and H4 (open symbols) in June-August (mean ± SD; years 1986–2009). Significant differences between the stations (paired t-test, p<0.05) are indicated in red. Dotted line indicates 5% threshold for including a phytoplankton group in the GLM analysis of the field survey study.

The copepods (17 females and 3 males per bottle, 12 replicates per treatment) were incubated in two alternative feeding regimes, with or without *N. spumigena* in the media, at 20°C. In the treatments without cyanobacteria, a monoculture of *B. submarina* (1061±87 µg C L^−1^; average ± SD) was used as a sole food, and in the treatments with cyanobacteria, *B. submarina* (971±208 µg C L^−1^) were mixed with *N. spumigena* (102±18 µg C L^−1^); this mixture approximates average *N. spumigena* contribution to the summer phytoplankton community in the northern Baltic proper (Figure 2). The bottles were incubated on a plankton wheel (1 rpm), with 16∶8 h light:dark cycle. Prior to the experimental incubation, the copepods were acclimatized under the same conditions for 36 h. At the end of the experiment, the copepods were filtered through a 120 µm mesh and examined under a stereo microscope. Live copepods were transferred to Eppendorf tubes and stored at −80°C for stomach content analysis using qPCR (quantitative real-time polymerase chain reaction) and oxidative status analysis. To get sufficient amount of material for these analyses, all material collected from consecutive replicates were pooled two and two within a treatment, giving 6 replicates for each treatment. Eggs and nauplii from each bottle were collected with a 48 µm mesh, stored overnight at 3°C in dark, and used to estimate EPR, EV% and development index (DI).

#### Sample preparation for molecular and biochemical assays

To determine amount of *N. spumigena* in the copepod gut content and to characterize oxidative status of the copepods, adult animals recovered from the incubation bottles were placed into microcentrifuge tubes with 0.7 mL phosphate buffer saline (PBS) and 100 µm glass beads and homogenized for 4 minutes using FastPrep with cooling. The tubes were thereafter centrifuged at 4°C for 5 min with 10 000×*g*.

#### Molecular diet analysis

For DNA extraction, 50 µL of the homogenate were used by mixing with 50 µL 20% Chelex [39]. These samples were heated at 105°C for 30 min, centrifuged and the supernatant was used to quantify *N. spumigena* using qPCR. The *Nodularia*-specific primers were used to amplify ∼200 bp fragment of *N. spumigena* 16S rDNA in the guts of copepods [15]. All qPCR reactions were performed in a final volume of 25 µL reaction mixture with a StepOne real-time cycler (Applied Biosystems) using the KiCqStart SYBR Green qPCR Ready Mix (Sigma). To prepare a standard curve of five step 10-fold dilutions (8.2 to 8.2×10^−4^ ng), DNA extracted from a culture of *N. spumigena* was used [40]; the linearity of the standard curve was high (R^2^>0.99), with amplification efficiency of 95–100%. As a reference sample, the feeding media containing *N. spumigena* but no copepods following the same procedure as for the test samples was used. A standard curve and no template controls (water) in triplicates were included in all runs. To check for non-specific products, DNA melt-curve analysis was performed after each qPCR experiment.

#### Oxidative stress biomarkers

To measure the intracellular soluble antioxidant capacity, the homogenized copepod samples were analyzed using oxygen radical absorption capacity (ORAC; µM trolox equivalents) assay [41]; see Protocol S1. To measure oxidative damage, the lipid peroxidation assay using QuantiChrom thiobarbituric acid reactive substances (TBARS; mol MDA Assay Kit; DTBA-100; BioAssay Systems, USA) was used following the manufacturer's directions. The ratio between ORAC and TBARS (ORAC:TBARS ratio) was used as a proxy for oxidative balance [32].

#### Reproductive state variables

To estimate copepod reproductive output, the female EPR, the EV% and early nauplial development (development index; DI) were used. To determine EPR, all eggs were counted and related to the number of live females in the corresponding bottle. For EV% measurements, ∼50 eggs per bottle were analyzed with TO-PRO-1 iodide staining [35]. The remaining eggs and nauplii were preserved with acidic Lugol's solution for calculating DI, which incorporates survival and metamorphosis success in copepods:
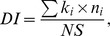
where *k_i_* is assigned stage value (in our study: 0 for egg, 1 for nauplii NI, and 2 for nauplii NII; no nauplii had developed beyond NII), *n_i_* number of copepods at that stage, and *NS* – total number of individuals [42]. All nauplii from the incubations (i.e., Lugol-preserved samples for DI-analysis, and nauplii hatched in the TO-PRO-stained samples) were included in the EV calculation. VEPR was estimated by multiplying EV and EPR.

### Long term data analysis

Zooplankton collected in the northern Baltic proper within SNMMP and SYVAB's marine monitoring program in Himmerfjärden Bay (Himmerfjärden Eutrophication Study; www.2.ecology.su.se) were used to study effects of various phytoplankton groups and physical factors on copepod growth and recruitment, with particular focus on the effects of cyanobacteria. As a proxy for copepod growth during early life stages, we used RNA:DNA ratio in the nauplii (feeding stages, NIII–NVI) of *Acartia* spp. (*A. bifilosa*, *A. tonsa* and *A. longiremis*) and *Eurytemora affinis*. In the study areas, *A. bifilosa* dominate in summer *Acartia* communities, contributing 62 to 94% on the long-term basis (E. Gorokhova, pers. obs.). Specifically, we considered that biotic (biovolumes of total phytoplankton, phytoplankton excluding filamentous cyanobacteria, and specific phytoplankton groups) and abiotic (North Atlantic Oscillation [NAO] indices, SST and salinity) variables integrate local environmental variability, and could define an adequate environmental framework for copepod growth.

#### Sampling

We used 34 and 36 samples for stations H4 and B1, respectively, collected every other week in July and August, i.e., when cyanobacteria are abundant in the study area, during 1999–2009, resulting in 3–4 samples per station and year. On each sampling occasion, zooplankton and phytoplankton were collected in concert; salinity and SST were measured by CTD (Meerestechnik Elektronik GmbH). Phytoplankton samples were collected as integrated hose samples, preserved and analyzed in the same way as in the summer field survey [33], [34]. Zooplankton samples were taken by vertical tows from near bottom to surface using a WP2 net (diameter 57 cm, 90 µm mesh size). From each tow, randomly selected zooplankton were preserved in bulk using RNA*later* and stored for 12 to 24 months at −20°C until the nucleic acid analysis [43].

#### RNA:DNA ratio

RNA and DNA contents were measured in nauplii (NIII–NVI) of *Acartia* spp. and *E. affinis*. The younger nauplial stages (NI–NII) were considered non-feeding [44] and thus not included in the analysis. Individual specimens were picked from the RNA*later* preserved samples, rinsed and transferred to Eppendorf tubes (5–7 ind. sample^−1^); the two copepod genera were treated separately. Nucleic acids were quantified with fluorometric high-range RiboGreen (Molecular Probes, Inc., Eugene, OR assay) using the same instrumentation as for RNA analysis in the summer field survey [37]. Mean standard curve slope ratio (mDNA/mRNA) was 1.87.

### Data analyses

The environmental data and phytoplankton biovolume data used in all analyses are available from www.smhi.se (SHARK database) and www.2.ecology.su.se/dbhfj/index.htm, and data on copepod growth- and reproduction-related variables are provided as Supporting Information (Summer field survey: Table S1; Laboratory experiment: Table S2; and Long-term data analysis: Table S3).

To evaluate effects of feeding environment with particular focus on cyanobacteria on copepod reproduction, growth and oxidative status, Generalized Linear Models (GLMs) with normal error structure and either identity or log-link function were applied using STATISTICA 10 (StatSoft, Inc., 2010). For all GLMs, the response variables and biovolume data were Box-Cox transformed to approach normal distribution. Akaike Information Criterion (AIC) was used to optimize the number and combination of predictive variables included. To validate the models, the Wald statistic was used to check the significance of the regression coefficient for each parameter, a likelihood ratio test was used to evaluate the statistical significance of including or not including each parameter and model goodness of fit was checked using deviance and Pearson χ^2^ statistics. Residual plots for each model were assessed visually to exclude remaining unattributed structure indicative of a poor model fit.

#### Summer field survey

GLMs were used to examine relationships between the dependent variables (reproductive/growth variables: VEPR, EPR, EV% and RNA) and the explanatory variables (biovolumes of filamentous cyanobacteria species [*Aphanizomenon* sp., *N. spumigena* and *Dolichospermum* spp.], autotrophs in each size fraction [2–5 µm; 5–15 µm; 15–30 µm and>30 µm], total phytoplankton biovolume, salinity and SST]. Copepod PL was also included as independent variable for RNA and EPR [45].

#### Laboratory experiment

Based on the positive effects of *N. spumigena* observed in the summer field survey and recent reports on high levels of antioxidants in cyanobacteria [46] as well as stimulation of antioxidant defenses in grazers feeding on hepatotoxic cyanobacteria [47], GLMs were used to test whether copepod reproductive output (EPR, EV%, VEPR and DI), antioxidant levels (ORAC), lipid peroxidation (TBARS), and oxidative balance (ORAC:TBARS ratio) were positively affected by grazing on *N. spumigena*. The amount of *Nodularia* in copepod guts determined by qPCR was used as a measure of grazing.

#### Long term data analysis

GLMs were used to examine relationships between the nauplial RNA:DNA ratio and biovolume of each phytoplankton group and abiotic variables that have been reported to affect zooplankton in the Baltic Sea (NAO, SST and salinity). Wilcoxon signed rank test was used to determine the significance of differences in nauplial RNA:DNA ratio between the stations (B1 and H4) and between the months (July and August) within the stations, whereas paired t-test was used to evaluate these differences in phytoplankton. The models were constructed for each taxa (*Acartia* spp. and *E. affinis*) and station (B1 and H4), because of the significant differences in RNA:DNA ratio between the species and differences in phytoplankton community structure between the stations. Only those phytoplankton groups that contributed>5% to the total phytoplankton biovolume were considered (Figure 2); these were: (1) dinoflagellates, (2) pooled group of small (<10 µm) unidentified flagellates and Prymnesiophyceae species, (3) diatoms, (4) *Mesodinium rubrum* (autotrophic ciliate), (5) nitrogen fixing filamentous Cyanobacteria, (6) Cryptophyceae, (7) large (>10 µm) Prymnesiophyceae, and (8) Prasinophyceae. We also included biovolumes of total phytoplankton and phytoplankton without cyanobacteria. The monthly NAO index values were taken from the Climate Prediction Center, Washington, DC (ftp://ftp.cpc.ncep.noaa.gov/wd52dg/data/indices/nao_index.tim). A high, positive winter NAO index (wNAO; December – March) indicates mild and rainy winters, while low, negative values occur during cold winters over Europe. A warmer winter is generally followed by a warmer spring (sNAO; March – May) with early onset of the growth season.

## Results

### Summer field survey

**Figure 3:**
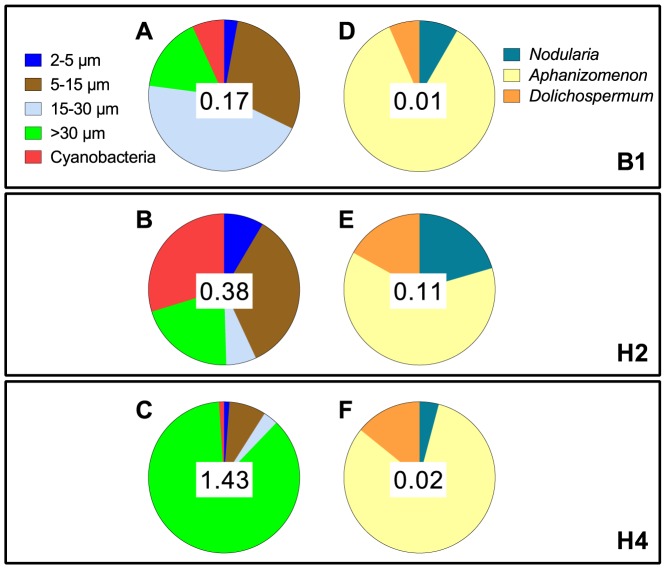
Composition of phytoplankton (A–C) and cyanobacterial (D–F) communities during the study period at: (A, D) stn B1, (B, E) stn H2, and (C, F) stn H4. Number in the middle of each pie chart indicates total biovolume (mm^3^ L^−1^) of the contributing categories.

Phytoplankton communities differed among the stations, with the highest total phytoplankton (1.43 mm^3^ L^−1^) and highest contribution of filamentous cyanobacteria (29%) observed at stns H4 and H2, respectively (Figure 3). All cyanobacterial communities dominated by *Aphanizomenon* sp. (62–85% of total cyanobacteria; Figure 3). There were significant, albeit weak, negative correlations between the cyanobacteria and the two largest size classes of phytoplankton, 15–30 µm and>30 µm (Pearson *r*: −0.29 and −0.36 respectively; *p*<0.05), and a significantly positive correlation between the cyanobacteria and the smallest phytoplankton size class (2–5 µm; Pearson *r*: 0.38; *p*<0.05), whereas no correlation was found for the intermediate size class (5–15 µm; Pearson *r*: 0.13; *p*>0.05). Among the cyanobacteria, *Aphanizomenon* sp. had the highest significant negative correlation with phytoplankton>15 µm (Pearson *r* = −0.38; *p*<0.05) and *N. spumigena* had a moderate positive correlation with phytoplankton 2–5 µm (Pearson *r* = 0.53; *p*<0.05).

**Figure 4:**
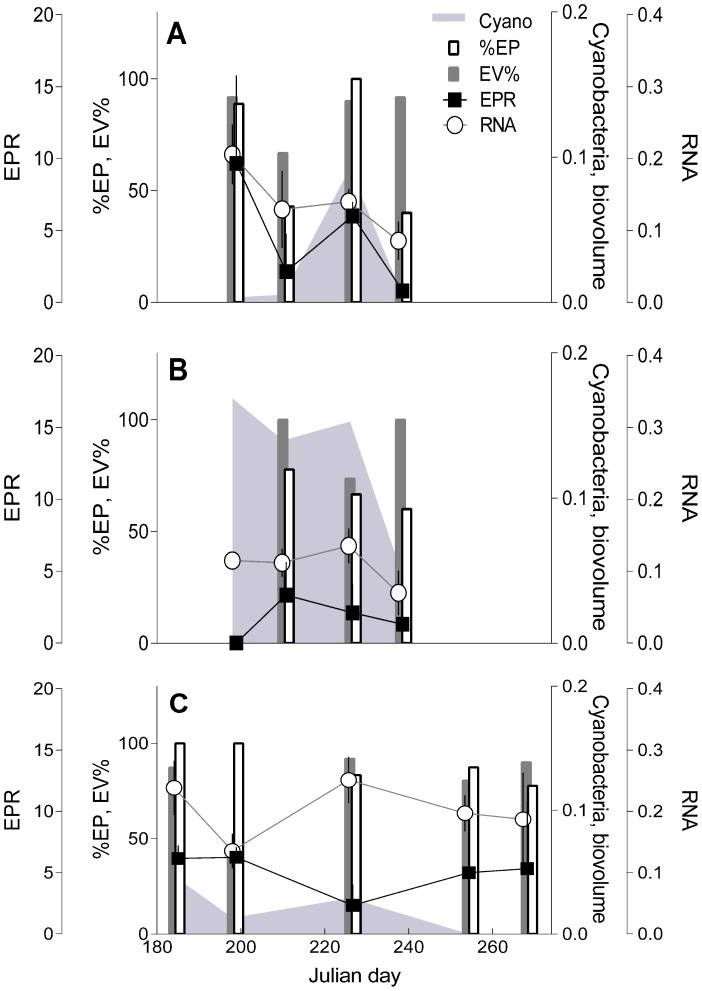
Seasonal dynamics of reproductive and growth variables in the copepod *Acartia tonsa* in relation to cyanobacteria bloom progression (biovolume, mm^3^ L^−1^) at stations B1 (A), H2 (B) and H4 (C). Egg production rate (EPR; eggs female^−1^ day^−1^), egg viability (EV%; % viable eggs), percentage of females producing eggs during 24-h incubation (%EP) and individual RNA content (µg ind^−1^).

The maximum values for EPR and individual RNA content were observed in the first half of July, whereas their minima occurred in the end of August. The opposite trend was observed for EV%, with generally low values in July and high in the end of August (Table S4, Figure 4). The GLMs indicated that *N. spumigena* was a significant positive predictors for all reproductive variables (EPR, EV% and VEPR) but not the RNA content (Table 1). *Aphanizomenon* sp., on the other hand, significantly affected only EPR (stns B1 and H4) and the effect was negative. No statistically significant model for EPR was found for stn H2, which had significantly lower EPR compared to stns B1 and H4 (Table 1). Significant positive relationships were observed between phytoplankton 15–30 µm and all response variables. Also, phytoplankton>30 µm was significantly positively related to VEPR and RNA, albeit the relationships were relatively weak (Table 1).

**Table 1:** GLM results of the summer field study.

Response variable	Stn	Explanatory variables	Beta	*p*	*R* ^2^	Adj. *R* ^2^	SE estimate
**EPR**	B1, H4*	*Nodularia*	124.5	<0.0001	0.39	0.35	3.29
		*Aphanizomenon*	−18.6	<0.0001			
		2–5 µm	42.1	0.001			
		15–30 µm	7.6	0.0001			
**EV%**	All	*Nodularia*	7.0	0.006	0.28	0.19	0.21
				0.008			
**VEPR**	All	*Nodularia*	7.6	<0.0001	0.39	0.35	3.29
		15–30 µm	6.4	0.034			
		>30 µm	1.3	<0.0001			
				0.004			
**RNA**	All	15–30 µm	2.1	<0.0001	0.64	0.62	0.04
		>30 µm	0.6	<0.0001			
		PL	0.1	<0.0001			
				0.001			

Response variables: egg production rate (EPR; eggs female^−1^ day^−1^), egg viability (EV%; % viable eggs), viable egg production rate (VEPR; viable eggs female^−1^ day^−1^), individual RNA content (µg ind^−1^). Explanatory variables: biovolume of different size fractions of phytoplankton and cyanobacteria species, copepod prosome length (PL) and station (B1: *n* = 28; H2: *n* = 29; and H4: *n* = 33). When a significant station effect was found, separate models were fitted for stations without significant differences. In EV% analysis, only females that produced eggs during incubation were included (*n* = 58).

*No significant model was found for stn H2.

### Laboratory experiment

In copepods, incubated in the presence of *N. spumigena* in the feeding media, amount of the cyanobacterium per stomach estimated by qPCR varied ∼3-fold (0.47 to 1.32 ng *Nodularia* DW ind.^−1^); none of the controls were positive (Table S4). The amount of *N. spumigena* per copepod had significant positive effects on all response variables tested, except EPR where it was negative (Table 2). The concurrent opposite effects on EPR and EV% resulted in no significant influence on VEPR (Table 2). Also, the significant positive effect of *N. spumigena* on ORAC in combination with negative but not significant effect on TBARS resulted in the significant positive effect on the ORAC:TBARS ratio (Table 2).

**Table 2:** GLM results of the experimental data.

Response variable	Estimate	Wald stat	*p*
EPR	−0.686	13.73	**0.0002**
EV%	1.560	10.643	**0.0011**
VEPR	−0.293	1.783	0.1817
DI	1.687	4.279	**0.0385**
ORAC	0.004	7	**0.0101**
TBARS	−0.814	2.391	0.1220
ORAC:TBARS	0.00003	4.299	**0.0381**

All models tested effects of grazing on *Nodularia spumigena* measured as amount of the cyanobacterium in the copepod guts (ng *Nodularia* DW ind^−1^) on reproductive output (EPR, EV%, and VEPR), nauplial development (DI), antioxidant (ORAC) and lipid peroxidation (TBARS) levels as well as the resulting oxidative balance (ORAC:TBARS ratio). Abbreviations and units for all response variables as in Tables 1 and 2. Significant models are in bold face.

### Long term data

**Figure 5:**
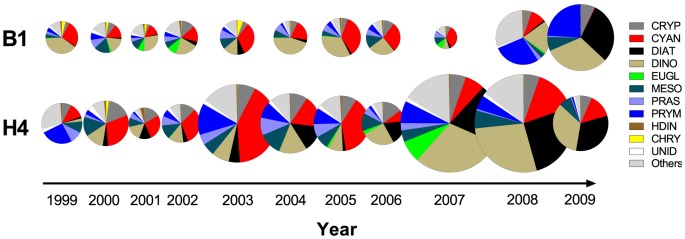
Long-term (1999–2009) dynamics of phytoplankton community structure at stations B1 (A) and H4 (B). Data are averages for July and August, weeks 27 to 35 (e.g., period of the summer cyanobacterial bloom, *n* = 3 or 4). Circle diameter is proportional to the average total phytoplankton biovolume (mm^3^ L^−1^) observed during the same period (0.43±0.15, *n* = 70). Abbreviations: CHLO – Chlorophyceae, CHRY – Chrysophyceae, CRYP – Cryptophyceae, CYAN – Cyanophyceae, DIAT – Diatoms, DINO – Dinophyceae, EUGL – Euglenophyceae, MESO – *Mesodinium rubrum* (Myrionecta rubrum), PRAS – Prasinophyceae, PRYM – Prymnesiophyceae, UNID – unidentified flagellates, and HDIN – heterotrophic dinoflagellates.

**Figure 6:**
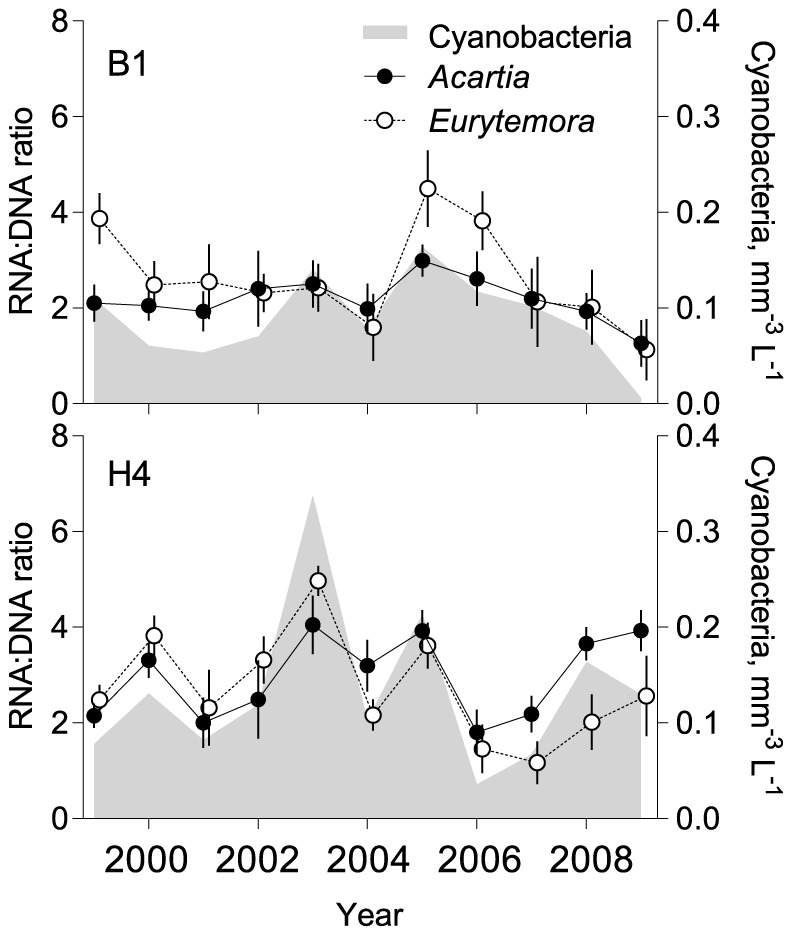
Long-term (1999–2009) dynamics of RNA:DNA ratio (mean ± SD, *n* = 3 or 4) in the nauplii (NIII-NVI) of copepods *Acartia* spp. and *Eurytemora affinis* in relation to cyanobacteria biovolume at stations B1 (A) and H4 (B). Data are averages for July and August, weeks 27 to 35.

Significant differences were found between the stations in terms of the phytoplankton community structure over the years (Figures 2 and 5). Cyanobacteria contribution to the total phytoplankton biovolume during the bloom period (July-August) varied from less than 1%, with the lowest values observed in 2009 at both stations to 40% at B1 and 45% at H4, in 2007 and 2003, respectively. Other important phytoplankton groups were diatoms (up to 39% of the total biovolume), dinoflagellates (up to 47%) and prymnesiophyceans (up to 40%). Total phytoplankton biovolume also varied both over time and between the stations, reaching its peak in 2008–2009 and 2007–2008 at B1 and H4, respectively (Figure 5), with significantly higher values at stn H4 compared to stn B1 (Wilcoxon signed rank; *p*<0.0017, *n* = 33). Moreover, the nauplial RNA:DNA ratio was also significantly higher at stn H4 than at stn B1 (Wilcoxon signed rank; *Acartia* spp.: *p*<0.004; *E. affinis*: *p*<0.002). Therefore, to describe responses of nauplial RNA:DNA ratio (Figure 6) to variations in phytoplankton groups over time (Figure 5), the GLMs were fit for each station separately.

For *Acartia* spp., only models based on filamentous cyanobacteria, diatoms, cryptophyceans, and abiotic parameters (SST, salinity, sNAO and wNAO) were significantly predictive. For *E. affinis*, only models incorporating total phytoplankton, filamentous cyanobacteria, diatoms and SST were significant (Table 3). Thus, in both species, the RNA:DNA ratio was significantly positively associated with the amount of cyanobacteria (Figure 6); moreover, parameter estimates indicated that these effects were the strongest in all models that included cyanobacteria (Table 3). Effects of other phytoplankton groups (cryptophyceans, total phytoplankton and diatoms) were all negative (Table 3). At stn B1, these groups correlated significantly negatively with cyanobacteria (Pearson *r*; −0.56, −0.44 and −0.62 for cryptophyceans, total phytoplankton and diatoms, respectively; Table S5). At stn H4, the effects of diatoms were also negative, but no significant correlations between the cyanobacteria and other phytoplankton groups were observed (Table S5).

**Table 3:** Best-fit GLMs relating RNA:DNA ratio in the copepod nauplii (*Acartia* spp. and *E. affinis*) to biovolumes of total phytoplankton (TotPhyto) and specific phytoplankton groups as well as climatic factors (SST, salinity and NAO indices) in the long-term dataset for stations B1 and H4.

Station (*n*)	Phytoplankton group tested	Explanatory variables	Estimate	Wald statistics	*p*
*Acartia* spp.					
**B1**	Cyanobacteria	Cyanobacteria	3.788	170.892	<0.0001
(36)		SST	0.045	21.118	<0.0001
	Cryptophyceans	Cryptophyceans	−7.002	18.845	<0.0001
		SST	0.149	36.445	<0.0001
**H4**	Cyanobacteria	Cyanobacteria	1.583	9.736	0.0018
(34)		wNAO	0.566	17.104	<0.0001
		sNAO	−0.304	15.284	<0.0001
		Salinity	1.240	13.593	0.0003
	Diatoms	Diatoms	−0.820	5.838	0.0157
		wNAO	0.603	18.495	<0.0001
		sNAO	−0.376	22.139	<0.0001
		Salinity	1.189	11.680	0.0007
*Eurytemora affinis*					
**B1**	TotPhyto	TotPhyto	−1.326	9.077	0.003
(36)		sNAO	−0.346	26.641	<0.0001
	Cyanobacteria	Cyanobacteria	5.164	22.059	<0.0001
		sNAO	−0.142	5.718	0.0282
	Diatoms	Diatoms	−3.248	4.796	0.0295
		sNAO	−0.152	13.031	0.0012
**H4**	Cyanobacteria	Cyanobacteria	2.611	47.899	<0.0001
(34)		wNAO	0.327	10.008	0.0016
		Salinity	−0.778	6.870	0.0087
	Diatoms	Diatoms	−1.646	18.088	<0.0001
		sNAO	−0.400	24.733	<0.0001
		wNAO	0.745	32.529	<0.0001
		Salinity	−1.384	16.06	<0.0001

sNAO and wNAO are NAO indices averaged for spring (March – May) and winter (December – March), respectively; *n* – number of observations.

Among the abiotic parameters, wNAO and sNAO indices were repeatedly indicated as significant, with positive effects of wNAO and negative of sNAO (Table 3). In addition, salinity had positive effects on RNA:DNA ratio in *Acartia* spp. and negative in *E. affinis*, whereas SST effect was significant only for *Acartia* spp. at B1 (Table 3).

## Discussion

Both positive and negative effects of Baltic filamentous cyanobacteria on copepod biochemical and physiological responses were observed in the field and laboratory settings (Table S6). Moreover, these effects were species-specific and differed, in some cases, among the studies. However, contrary to the widely reported harmful effects of these cyanobacteria on the copepod reproduction [2], no negative effects on the net reproductive output, i.e. viable egg production, were found. In fact, viable egg production in *A. tonsa* was positively related to *N. spumigena* abundance in the field (Table 1). Although no effect of *N. spumigena* on viable egg production was observed in the laboratory experiment with *A. bifilosa*, the development of nauplii (non-feeding stages) was significantly advanced if their mothers were feeding on the cyanobacterium, thus effectively increasing recruitment (Table 2). Also, the long term data analysis also identified diazotrophic filamentous cyanobacteria as significant positive growth predictors for the copepod nauplii (Table 3).

Specific mechanisms behind the observed stimulating and suppressing effects of diazotrophic filamentous cyanobacteria on copepod reproduction and early development are largely unknown. We suggest three, not mutually exclusive, pathways involved in stimulating effects of cyanobacteria on copepod reproduction: (1) direct increase of macro- and micronutrient intake by copepods that use cyanobacteria as an additional food source; (2) supplementing copepod's diet with phytochemicals, such as polyphenols, vitamins and various antioxidants, that may enhance physiological responses [46], including hormesis effect, which is a generally favorable biological response to low exposures of toxins and other stressors [47]; and (3) indirect increase of nutrient intake by stimulation of the microbial loop through cyanobacterial exudates, resulting in increase of bacterio- and nanoplankton prey for smaller copepods [48]. The qPCR-based gut content analysis in the laboratory experiment confirmed that the copepods were actively feeding on *N. spumigena* (Table S4), and thus, the observed variations in the reproductive variables are – at least partly – the result of the direct grazing. Some filamentous cyanobacteria could be nutritionally valuable for grazers, particularly in combination with other food sources [49]. Moreover, as pointed out by Jiang and colleagues [50], *“a putatively harmful alga is not always deleterious to grazers, and its ecological effects may be distinctly different during bloom and non-bloom periods”*. They found that toxicity and nutritional value of the dinoflagellate *Cochlodinium polykrikoides* went from deleterious to beneficial for *A. tonsa* when the algal density decreased. Cyanobacteria may contain complimentary nutrients and microelements, e.g. amino acids, antioxidants, vitamins, proteins, phosphorus and nitrogen [3], [49], [51], [52] that are of particular value for copepods. Nitrogen, for instance, is frequently depleted in the pelagia during summer in the Baltic Sea [53] and can thus be limiting for copepod reproduction [54]. Therefore, feeding on nitrogen fixing cyanobacteria may relax this limitation.

In addition to the positive effects on egg viability and nauplial development, the presence of *N. spumigena* increased the antioxidant defenses and improved oxidative status as indicated by the ORAC:TBARS ratio (Table 2). This is in contrast to other studies implicating hepatotoxic cyanobacteria as pro-oxidants for grazers [31]. However, the nature of cyanobacteria-induced oxidative stress is poorly understood; in fact, cyanobacteria produce dietary antioxidants that may directly improve antioxidative capacity of consumers [52]. Moreover, microcystins have been shown to increase activity of antioxidant enzymes in estuarine crabs [55]. The observed increase in antioxidant capacity may be indicative of the hormetic response to the cyanobacterial toxins and bioactive compounds, known as *The Xenohormesis Hypothesis* [56]. The latter has been suggested to be an evolutionary adaptation to sustain fitness in a changing environment, where the presence of a toxin in low concentrations acts as a signal for the organism to mobilize metabolic reserves to prepare itself for higher environmental stress [47], [56], [57]. Some of phytochemicals have evolved as toxins to intimidate grazers, triggering adaptive stress responses by, for instance, stimulating the production and/or activity of antioxidant enzymes in the consumer [56], [58]. Nodularins and microcystins produced by *N. spumigena* and *Dolichospermum* spp., respectively, could have contributed to the observed positive linkages between these cyanobacteria and copepod egg viability. One can speculate that an increase in the antioxidant capacity of a female triggered by a hepatotoxin and/or another secondary metabolite could enhance allocation of maternal antioxidants to the eggs, which improved embryonic and post-embryonic development.

The positive linkages between nauplial growth inferred from the RNA:DNA ratio and total diazotrophic filamentous cyanobacteria in the long term data set (Table 3) are not likely to be due to the grazing on cyanobacteria by the nauplii. Although some grazing on *N. spumigena* colonies by copepod nauplii has been observed [59], the pathway involving fueling of microbial communities is much more likely. During summer, when inorganic nitrogen is depleted, bloom-forming cyanobacteria are responsible for a significant proportion of N_2_-fixation in the Baltic Sea [60]–[63]. As much as one third of the fixed N_2_ leaks out as ammonium NH_4_^+^ from these cyanobacteria [62], [63] and is further utilized by other organisms, such as heterotrophic bacteria and picoautotrophs [64]. The microbial loop is also stimulated by dissolved organic matter (DOM) and detritus derived from cyanobacteria and utilized by bacteria [64], [65]. Nauplii and, to a lesser extent, copepodites, graze on pico- and bacterioplankton [48], [65], [66], which also support dietary nano- and microzooplankton, and these communities flourish in association with *N. spumigena* [67]. Therefore, N-leakage from diazotrophic cyanobacteria, which stimulate microbial prey, could explain the positive association between nauplial growth and filamentous cyanobacteria during summer blooms (Table 3). Remarkably, cyanobacteria were the only phytoplankton group positively related to the nauplial RNA:DNA ratio (Table 3, Figure 6). Although cryptophyceans, total phytoplankton and diatoms were all negatively associated with nauplial growth at stn B1 (Table 3), these negative effects may, at least partially, be explained by the negative cross-correlations between these groups and the cyanobacteria (Table S5); see also [68]. However, the negative effect of the diatoms on the nauplial growth at stn H4 cannot be explained by such correlation (Table S5). In this case, diatoms may have affected nauplii either directly, e.g., via deleterious effects [69], or by interspecific interaction with some other prey that was beneficial for growth. The observed effects of salinity (Table 3) are rather expected as *A. bifilosa* is a species of marine origin, whereas *E. affinis* is a brackish water copepod thriving in a broad salinity range [22]. Moreover, high genetic diversity of Baltic *E. affinis* [70] may have contributed to the varying responses to winter and spring NAO (Table 3).

While stimulation of the microbial food web is possible in the field, it cannot explain the positive effects of *N. spumigena* on copepod egg viability and non-feeding nauplial development in the experiment (Table 2), because the copepods were incubated in 0.2 µm filtered sea water, where bacteria were heavily reduced and flagellates and ciliates were largely eliminated. Hence, both the direct and indirect pathways are likely to be responsible for the positive relationships between *N. spumigena* and copepod reproduction observed in the summer field survey (Table 1). The microbial communities thriving in cyanobacterial bloom may nourish the copepods either via the microbial loop [65], [71] or as epibions on the ingested cyanobacterial colonies. Moreover, the mechanisms may differ between the cyanobacteria species as well as among zooplankton grazers.

Although the net effects of cyanobacteria on copepod reproduction (i.e., VEPR) appear either neutral or positive, there is some variation among the species and studies. For example, in the summer field survey, effects of *N. spumigena* on egg production in *A. tonsa* were positive (Table 1), whereas in the experimental study, this effect in *A. bifilosa* was negative, albeit these opposite responses resulted in no significant net effect on viable egg production (Table 2). This discrepancy between the laboratory experiment and the summer field survey suggest that variations in the feeding environment may be crucial for cyanobacteria-copepod interactions. In the field, *N. spumigena* had a significant positive correlation with biovolume of edible phytoplankton (15–30 µm), which was also a positive predictor for egg production; whereas these were not available for the copepods in the experiment. There might also be differences between the test species, *A. tonsa* (field survey) and *A. bifilosa* (experiment), in their sensitivity to the cyanobacterium effects and dependency on other food sources and environmental factors. In particular, *A. bifilosa* might have been avoiding to ingest larger filaments in the experiment due to food selection against this cyanobacterium [72]. This would effectively decrease food availability in the incubations as food (*Brachiomonas* with or without *Nodularia*) was provided at the same amount. As a result, lower food intake when feeding in mixtures containing cyanobacteria would translate into lower egg production, which was observed in this copepod species.

The observed differences among the cyanobacteria species could also be related to their relative abundance, morphology, nutritional value and/or biochemistry of toxins and metabolites. *N. spumigena* colonies do not form bundles, are not as rigid [73], and could, therefore, be easier to handle for mesozooplankton grazers than *Aphanizomenon* sp., which was found to negatively affect EPR (Table 1), and which has been reported to impair copepod reproduction to a higher extent than *N. spumigena* [29]. These cyanobacteria species also differ in their abundance (Figure 3) and nutritional value [49], [51], including the value of their microbial epibionts [67], [74]. *Aphanizomenon* sp. has lower colonization by heterotrophic bacteria compared to *N. spumigena* [59], [63], which hosts a lucrative microenvironment for microorganisms [62], [67], [75]. This was also supported by significant positive correlation between *N. spumigena* and phytoplankton 2–5 µm observed in the summer field survey. This size fraction might be more N-sufficient than larger phytoplankton and thereby provide the more balanced food for the copepods. In line with this, significant positive effects of the phytoplankton 2–5 µm on the copepod reproductive variables were observed (Table 1). The highest egg production has been reported to occur when the diet consisted of alternative food with small contribution of cyanobacteria, mainly *N. spumigena* [29]; this resembles the *in situ* feeding conditions in our field study. The negative correlation between *Aphanizomenon* sp. and EPR (Table 2) could also be a result of poor feeding conditions for the copepods, due to scarcity of edible phytoplankton during the cyanobacteria bloom. Indeed in the field survey, *Aphanizomenon* sp., among the filamentous cyanobacteria, had the highest significant negative correlation with phytoplankton>15 µm. For *A. tonsa*, the optimal food size is 2–5% of the prosome length [76], which implies that optimal food size for the females sampled here would be 15–40 µm, explaining the observed positive relationships between phytoplankton 15–30 µm and growth related variables (RNA content and EPR; Table 1). Moreover, station had a significant influence on egg production, indicating presence of other factors, not accounted for in our analysis (Table 1).

In conclusion, our results indicate that summer blooms of diazotrophic filamentous cyanobacteria in the Baltic Sea could be important for copepod growth and reproduction by providing complementary food, supporting high antioxidant levels and fueling growth of microbial prey. In particular, *N. spumigena*, which are the most conspicuous bloom forming toxic filamentous cyanobacteria that build surface accumulations [20], is grazed upon and have positive effects on copepod recruitment and growth (Table S6). This was however not the case for the most abundant cyanobacterium, *Aphanizomenon* sp., which appears to decrease total egg production, with, however, neutral effects on the production of viable eggs. Given that dominant Baltic zooplankters are selectively feeding on non-toxic cyanobacteria or can avoid cyanobacterial filaments altogether [72], it is possible that by supporting growth and recruitment in grazer populations, these cyanobacteria may gain a competitive advantage over other phytoplankton [77]. In this case, the effects of cyanobacteria on grazers will be highly system-specific, depending on evolutionary trajectories of the species and populations in question. Our results, together with findings reporting high incorporation of diazotrophic nitrogen in pelagic and benthic food webs in this and other systems experiencing blooms of these cyanobacteria [78], [79], have important implications for understanding impacts of these blooms on secondary, and, ultimately, fish production in the Baltic Sea. Further investigations on the associated ecological and evolutionary trade-offs behind these interactions are needed, if we are to understand and manage eutrophication and fishery in this system.

## Supporting Information

Table S1 ***Acartia tonsa*** **: Data on reproductive and growth variables obtained in the summer field survey conducted in July - September 2007, at three coastal stations (B1, H2 and H4) in the northern Baltic proper.** Variables measured: EPR (egg production rate), VEPR (viable egg production rate), RNA (individual ribonucleic acid content), and PL (prosome length).
(XLSX)

Table S2 ***Acartia bifilosa*** **: Data on feeding, reproduction and nauplii development obtained in the laboratory experiment conducted in August 2010, in the Storfjärden Bay, Western Finland.** Variables measured: EPR (egg production rate), VEPR (viable egg production rate), RNA (individual ribonucleic acid content), and PL (prosome length).
(XLSX)

Table S3 ***Acartia*** **sp. and** ***Eurytemora affinis*** **: Long-term data (mean ± SD; period 1999–2009) on RNA:DNA ratio in nauplii at stns B1 and H4 in the northern Baltic proper.** The mean values are based on 3–4 replicate samples analyzed per year (July-August).
(XLSX)

Table S4 **Growth and reproduction indices observed in the (A) field survey, (B) experimental study, and (C) long-term dataset.** Data are presented as means with their standard deviations; abbreviations as in Tables S1 and S2.
(DOC)

Table S5 **Pearson** ***r*** **for correlations among phytoplankton groups at stn B1 and stn H4.** Significant correlations are in bold face (*p*<0.05). Abbreviations: CHLO – Chlorophyceae, CHRY – Chrysophyceae, CRYP – Cryptophyceae, CYAN – Cyanophyceae, DIAT – Diatomea, DINO – Dinophyceae, EUGL – Euglenophyceae, MESO – *Mesodinium rubrum* (*Myrionecta rubrum*), PRAS – Prasinophyceae, PRYM – Prymnesiophyceae, UNID – unidentified flagellates, and HDIN – heterotrophic dinoflagellates.
(DOC)

Table S6 **Summary of the observed effects linking filamentous cyanobacteria to reproductive output, growth indices and oxidative status in copepods.** Abbreviations as in Tables S1 and S2.
(DOC)

Protocol S1 **Oxidative stress biomarkers.**
(DOCX)
