# Supplementary material for: Bloom-Forming Cyanobacteria Support Copepod Reproduction and Development in the Baltic Sea
Source: PLoS One. 2014 Nov 19;9(11):e112692. doi: 10.1371/journal.pone.0112692 (PMC4237358; doi:10.1371/journal.pone.0112692)
Supplement: Table S4 — Growth and reproduction indices observed in the (A) field survey, (B) experimental study, and (C) long-term dataset. Data are presented as means with their standard deviations; abbreviations as in Tables S1 and S2. (DOC) [file pone.0112692.s004.doc]

**PLoS One │ Supporting Information**

**Bloom-forming cyanobacteria support copepod reproduction and development in the Baltic Sea**

Hogfors, Motwani, Hajdu, El-Shehawy, Holmborn, Vehmaa, Engström-Öst, Brutemark and Gorokhova

**Table S4**. Growth and reproduction indices observed in the (A) field survey, (B) experimental study, and (C) long-term study. Data are presented as means with their standard deviations; abbreviations: egg production rate (EPR; eggs female^-1^ day^-1^), egg viability (EV; % viable eggs), viable egg production rate (VEPR; viable eggs female^-1^ day^-1^), individual RNA content (µg ind^-1^), development index (DI), oxidative balance (ORAC:TBARS ratio) and copepod gut-content (ng *N. spumigena* DW ind^-1^). The experimental treatments included those with or without *N. spumigena* (*Nodularia* and Control, respectively).

| Sampling date,  period or treatment | VEPR | EPR | EV | RNA | RNA:DNA | DI | ORAC | TBARS | ORAC: TBARS | *Nodularia*,  gut content |
| --- | --- | --- | --- | --- | --- | --- | --- | --- | --- | --- |
| **Summer field survey** | | | | | | | | | | |
| 4 July | 5.3±1.5 | 6.2±1.2 | 87±20 | 0.24±0.05 |  |  |  |  |  |  |
| 18 July | 6.7±6.3 | 8.0±5.8 | 75±32 | 0.18±0.05 |  |  |  |  |  |  |
| 30 July | 2.6±2.7 | 2.8±2.6 | 90±32 | 0.12±0.04 |  |  |  |  |  |  |
| 15 Aug | 2.1±1.9 | 2.6±2.3 | 83±21 | 0.18±0.06 |  |  |  |  |  |  |
| 27 Aug | 1.0±1.2 | 1.1±1.3 | 97±11 | 0.08±0.03 |  |  |  |  |  |  |
| 12 Sep | 4.0±3.3 | 5.0±3.8 | 80±22 | 0.20±0.03 |  |  |  |  |  |  |
| 26 Sep | 5.1±5.9 | 5.3±5.9 | 90±19 | 0.19±0.08 |  |  |  |  |  |  |
|  |  |  |  |  |  |  |  |  |  |  |
| **Experiment** | | | | | | | | | | |
| Control | 6.9±3.0 | 11.0±4.6 | 64±12 |  |  | 0.14±0.11 | 212±8 | 0.51±0.06 | 425±68 | 0±0 |
| *Nodularia* | 6.8±3.2 | 8.8±3.2 | 75±19 |  |  | 0.20±0.09 | 222±7 | 0.43±0.09 | 543±150 | 1.1±0.3 |
|  |  |  |  |  |  |  |  |  |  |  |
| **Long-term study (1999-2009)** | | | | | | | | | | |
| *Acartia* spp |  |  |  |  | 2.52 ± 0.76 |  |  |  |  |  |
| *Eurytemora affinis* |  |  |  |  | 2.65 ± 0.97 |  |  |  |  |  |
