# Supplementary material for: Bloom-Forming Cyanobacteria Support Copepod Reproduction and Development in the Baltic Sea
Source: PLoS One. 2014 Nov 19;9(11):e112692. doi: 10.1371/journal.pone.0112692 (PMC4237358; doi:10.1371/journal.pone.0112692)
Supplement: Table S5 — Pearson r for correlations among phytoplankton groups at stn B1 and stn H4. Significant correlations are in bold face (p<0.05). Abbreviations: CHLO – Chlorophyceae, CHRY – Chrysophyceae, CRYP – Cryptophyceae, CYAN – Cyanophyceae, DIAT – Diatomea, DINO – Dinophyceae, EUGL – Euglenophyceae, MESO – Mesodinium rubrum (Myrionecta rubrum), PRAS – Prasinophyceae, PRYM – Prymnesiophyceae, UNID – unidentified flagellates, and HDIN – heterotrophic dinoflagellates. (DOC) [file pone.0112692.s005.doc]

**PLoS One │ Supporting Information**

**Bloom-forming cyanobacteria support copepod reproduction and development in the Baltic Sea**

Hogfors, Motwani, Hajdu, El-Shehawy, Holmborn, Vehmaa, Engström-Öst, Brutemark and Gorokhova

**Table S5.** Pearson *r* for correlations among phytoplankton groups at stn B1 and stn H4. Significant correlations are in bold face (*p* < 0.05). Abbreviations: CHLO – Chlorophyceae, CHRY – Chrysophyceae, CRYP – Cryptophyceae, CYAN – Cyanophyceae, DIAT – Diatomea, DINO – Dinophyceae, EUGL – Euglenophyceae, MESO – *Mesodinium rubrum* (*Myrionecta rubrum*), PRAS – Prasinophyceae, PRYM – Prymnesiophyceae, UNID – unidentified flagellates, and HDIN – heterotrophic dinoflagellates.

Stn B1

| **B** | CHLO | CHRY | CRYP | CYAN | DIAT | DINO | EUGL | MESO | PRAS | PRYM | UNID |
| --- | --- | --- | --- | --- | --- | --- | --- | --- | --- | --- | --- |
| CHRY | 0.13 |  |  |  |  |  |  |  |  |  |  |
| CRYP | 0.05 | **-0.50** |  |  |  |  |  |  |  |  |  |
| CYAN | 0.25 | 0.32 | **-0.56** |  |  |  |  |  |  |  |  |
| DIAT | -0.15 | -0.23 | **0.65** | **-0.62** |  |  |  |  |  |  |  |
| DINO | -0.10 | **-0.37** | **0.54** | **-0.48** | **0.75** |  |  |  |  |  |  |
| EUGL | -0.29 | -0.16 | 0.02 | -0.22 | -0.24 | **-0.44** |  |  |  |  |  |
| MESO | -0.06 | 0.08 | **0.38** | **-0.49** | **0.47** | 0.06 | -0.19 |  |  |  |  |
| PRAS | 0.17 | 0.33 | -0.27 | **0.45** | **-0.55** | **-0.66** | -0.02 | 0.18 |  |  |  |
| PRYM | 0.25 | **-0.36** | **0.66** | **-0.54** | **0.64** | **0.70** | -0.27 | 0.20 | **-0.47** |  |  |
| UNID | 0.06 | **0.56** | **-0.43** | 0.19 | **-0.65** | **-0.56** | 0.11 | 0.14 | **0.64** | **-0.40** |  |
| HDIN | -0.13 | 0.07 | -0.21 | 0.27 | **-0.59** | -0.26 | **0.44** | **-0.43** | 0.03 | **-0.46** | **0.45** |

**Stn H4**

|  | CHLO | CHRY | CRYP | CYAN | DIAT | DINO | EUGL | MESO | PRAS | PRYM | UNID |
| --- | --- | --- | --- | --- | --- | --- | --- | --- | --- | --- | --- |
| CHRY | -0.25 |  |  |  |  |  |  |  |  |  |  |
| CRYP | 0.30 | **-0.53** |  |  |  |  |  |  |  |  |  |
| CYAN | 0.19 | 0.04 | 0.02 |  |  |  |  |  |  |  |  |
| DIAT | 0.27 | -0.28 | **0.54** | -0.17 |  |  |  |  |  |  |  |
| DINO | 0.20 | -0.24 | 0.27 | -0.24 | **0.81** |  |  |  |  |  |  |
| EUGL | **0.46** | -0.08 | -0.05 | -0.27 | -0.01 | 0.06 |  |  |  |  |  |
| MESO | 0.12 | -0.04 | **0.48** | -0.12 | **0.48** | 0.10 | -0.23 |  |  |  |  |
| PRAS | 0.23 | 0.06 | -0.14 | 0.24 | **-0.48** | **-0.60** | -0.05 | 0.14 |  |  |  |
| PRYM | 0.06 | -0.32 | **0.39** | -0.22 | 0.33 | **0.36** | -0.14 | 0.10 | -0.08 |  |  |
| UNID | 0.26 | 0.24 | 0.08 | 0.30 | -0.26 | -0.34 | 0.06 | 0.32 | **0.46** | -0.17 |  |
| HDIN | -0.28 | 0.10 | -0.19 | -0.05 | **-0.51** | **-0.36** | 0.13 | **-0.42** | 0.02 | -0.27 | 0.13 |
