# Supplementary material for: Bloom-Forming Cyanobacteria Support Copepod Reproduction and Development in the Baltic Sea
Source: PLoS One. 2014 Nov 19;9(11):e112692. doi: 10.1371/journal.pone.0112692 (PMC4237358; doi:10.1371/journal.pone.0112692)
Supplement: Table S6 — Summary of the observed effects linking filamentous cyanobacteria to reproductive output, growth indices and oxidative status in copepods. Abbreviations as in Tables S1 and S2. (DOC) [file pone.0112692.s006.doc]

**PLoS One │ Supporting Information**

**Bloom-forming cyanobacteria support copepod reproduction and development in the Baltic Sea**

Hogfors, Motwani, Hajdu, El-Shehawy, Holmborn, Vehmaa, Engström-Öst, Brutemark and Gorokhova

**Table S6.** Summary of the observed effects linking filamentous cyanobacteria to reproductive output, growth indices and oxidative status in copepods. Reproductive variables included egg production (EPR), egg viability (EV%), viable egg production (VEPR), development index (DI); RNA-based growth indices were female RNA content and nauplii RNA:DNA ratio; and oxidative status was inferred from ORAC and TBARS levels and their ratio. Plus and minus signs denote a significant (<0.05) positive and negative effect, respectively.

| | **Summer field survey** *(A. tonsa)* | | | | --- | --- | --- | | *N. spumigena* | VEPR | + | | EPR | + | | EV% | + | | *Aphanizomenon* sp. | EPR | – | | **Experiment** (*A. bifilosa*) | | | | *N. spumigena* | ORAC | + | | ORAC:TBARS | + | | EPR | – | | EV% | + | | DI | + | | **Long term study** (nauplii of *Acartia* spp. and *E. affinis*) | | | | Total filamentous cyanobacteria | RNA:DNA | + | |
| --- | --- | --- | --- | --- | --- | --- | --- | --- | --- | --- | --- | --- | --- | --- | --- | --- | --- | --- | --- | --- | --- | --- | --- | --- | --- | --- | --- | --- | --- | --- | --- | --- | --- |
