# Supplementary material for: Bloom-Forming Cyanobacteria Support Copepod Reproduction and Development in the Baltic Sea
Source: PLoS One. 2014 Nov 19;9(11):e112692. doi: 10.1371/journal.pone.0112692 (PMC4237358; doi:10.1371/journal.pone.0112692)
Supplement: Protocol S1 — Oxidative stress biomarkers. (DOCX) [file pone.0112692.s007.docx]

**PLoS One │ Supporting Information**

**Bloom-forming cyanobacteria support copepod reproduction and development in the Baltic Sea**

Hedvig Hogfors, Nisha H. Motwani, Susanna Hajdu, Rehab El-Shehawy, Towe Holmborn, Anu Vehmaa, Jonna Engström-Öst, Andreas Brutemark and Elena Gorokhova

**Protocol S1. Oxidative stress biomarkers**

*TBARS Assay*. Lipid peroxidation was measured in 200 µL homogenate mixed 1:1 (v/v) with ice-cold trichloroacetic acid; PPB was used as a blank. The mixture was incubated on ice for 5 min and centrifuged at 9300 × g for 5 min. Reaction solution (100 µL of 83 mM thiobarbituric acid (TBA) in glacial acetic acid mixed with 1.5 M NaOH (1:1 v/v), pH 3.5) was added to 100 µL of supernatant and incubated in a boiling water bath for 1 h. After cooling, 200 µL 1-butanol: pyridin (15:1 v/v) mixture were added to all samples and standards. Fluorescence was measured in the organic phase at excitation/emission wavelengths of 540/590 nm. Concentrations were derived from a standard curve of 1,1,3,3-tetramethoxypropane (malonaldehyde acid; MDA). The results are reported in pM MDA equivalents ind^-1^.

*ORAC assay*. The ORAC was measured using the modified ORAC_FL_ method with fluorescein (Fluka; 79.6 nM well^-1^) as a fluorescent probe, 2,29- azobis (2-amidinopropane) dihydrochloride (AAPH; Sigma–Aldrich; 23 mM well^-1^) as a peroxyl radical source, and Trolox (Sigma–Aldrich; 21.7 µM well^-1^) as a standard. For each assay, 4 mL of the homogenate were brought to 20 mL with PPB and used to measure ORAC; the values were expressed in trolox-equivalents, in µg ind. ^-1^.
